# African Swine Fever Virus CD2v Protein Induces β-Interferon Expression and Apoptosis in Swine Peripheral Blood Mononuclear Cells

**DOI:** 10.3390/v13081480

**Published:** 2021-07-28

**Authors:** Sabal Chaulagain, Gustavo A. Delhon, Sushil Khatiwada, Daniel L. Rock

**Affiliations:** 1 Department of Pathobiology, College of Veterinary Medicine, University of Illinois at Urbana-Champaign, Urbana, IL 61802, USA; sabal@illinois.edu (S.C.); ksushil1@illinois.edu (S.K.); 2 School of Veterinary Medicine and Biomedical Sciences, Nebraska Center for Virology, University of Nebraska-Lincoln, Lincoln, NE 68583, USA; gdelhon3@unl.edu

**Keywords:** African swine fever virus, CD2v, interferon-β, NF-κB, CD58, lymphocyte, macrophage, apoptosis, pathogenesis

## Abstract

African swine fever (ASF) is a hemorrhagic disease of swine characterized by massive lymphocyte depletion in lymphoid tissues due to the apoptosis of B and T cells, a process likely triggered by factors released or secreted by infected macrophages. ASFV CD2v (EP402R) has been implicated in viral virulence and immunomodulation in vitro; however, its actual function(s) remains unknown. We found that CD2v expression in swine PK15 cells induces NF-κB-dependent IFN-β and ISGs transcription and an antiviral state. Similar results were observed for CD2v protein treated swine PBMCs and macrophages, the major ASFV target cell. Notably, treatment of swine PBMCs and macrophages with CD2v protein induced apoptosis. Immunoprecipitation and colocalization studies revealed that CD2v interacts with CD58, the natural host CD2 ligand. Additionally, CD58 knockdown in cells or treatment of cells with an NF-κB inhibitor significantly reduced CD2v-mediated NF-κB activation and IFN-β induction. Further, antibodies directed against CD2v inhibited CD2v-induced NF-κB activation and IFN-β transcription in cells. Overall, results indicate that ASFV CD2v activates NF-κB, which induces IFN signaling and apoptosis in swine lymphocytes/macrophages. We propose that CD2v released from infected macrophages may be a significant factor in lymphocyte apoptosis observed in lymphoid tissue during ASFV infection in pigs.

## 1. Introduction

African swine fever (ASF) is an acute viral hemorrhagic disease of domestic swine with mortality rates approaching 100%. Devastating ASF outbreaks and continuing epidemics starting in the Caucasus region and now in the Russia Federation, Europe, China and other parts of Southeast Asia (2007 to date) highlight its significance.

ASFV, the sole member of the Asfarviridae (Asfar, African swine fever, and related viruses), is a large, enveloped and genetically complex virus containing a double-stranded DNA genome of approximately 190 kilobase pairs, which encodes for over 170 proteins. Aspects of genome structure and replication strategy are shared between ASFV and other large dsDNA viruses, most notably poxviruses [1].

ASFV is the only known DNA arbovirus. In sub-Saharan Africa, the virus is maintained in a sylvatic cycle between wild swine (warthogs and bushpigs) and argasid ticks of the genus Ornithodoros. Unlike domestic swine, wild swine infected with ASFV are generally asymptomatic and show low viremias. Most adult warthogs in ASFV enzootic areas are seropositive and persistently infected. ASFV persistently infects ticks from which ASFV can be isolated years post-infection [1].

ASF in domestic pigs occurs in several forms, ranging from highly lethal (100% mortality) to subclinical. Hemostatic and hemodynamic changes (hemorrhage, edema, ascites and shock) resulting from intravascular activation of coagulation are observed in pigs infected with highly virulent ASFV strains [2,3,4]. ASFV infects cells of the mononuclear-phagocytic system, including highly differentiated fixed-tissue macrophages and specific lineages of reticular cells, and highly virulent strains induce extensive damage in affected tissues [5,6]. The ability of ASFV to replicate and induce marked cytopathology in these cell types in vivo appears to be a critical factor in ASFV virulence. A characteristic of acute ASF is the severe lymphoid tissue destruction and massive lymphocyte depletion observed in infected pigs. As lymphocytes do not support ASFV replication, factors released or secreted by infected macrophages have been implicated in triggering lymphocyte apoptosis [7,8,9,10,11]. However, viral and host factors responsible are poorly understood.

Macrophages play a central role in development of both innate and adaptive immune responses [12]. ASFV infection of monocytes and macrophages has been shown to alter the expression and secretion of various cytokines, including IFNs, TNF, IL-1 and TGF-β, and modulate apoptosis [5,10,11,13,14,15,16]. Uncharacterized soluble factors released by ASFV-infected macrophages inhibit proliferation of swine lymphocytes in response to lectins in vitro [17].

ASFV CD2v (EP402R) is a glycoprotein with homology to host the adhesion molecule, CD2, which is expressed on T and NK cells [18,19,20] and involved in host immunomodulation, virulence and induction of protective immune responses [21,22,23]. The role of CD2v in ASFV virulence is not clearly understood. Infection of pigs with a CD2v deletion mutant virus resulted in different infection phenotypes depending on the parental virus strain. CD2v deletion in the European isolate BA71 resulted in virus attenuation [22]. Additionally, *CD2v* gene mutations have been observed in other attenuated ASFV strains [24] further suggesting a role of CD2v in virus virulence. In contrast, deletion of *CD2v* from virulent strains Malawi and Georgia 2007, while affecting aspects of viral pathogenesis, did not significantly affect viral virulence [21,25]. Although non-essential for viral replication in pigs, *CD2v* is necessary for virus replication in the tick midgut and for generalization of infection in the tick [26,27].

The ASFV CD2v contains all the domains present in cellular CD2 and some of the residues involved in binding to its natural ligand, the cell adhesion molecule CD58/LFA-3 [18,19]. Interaction of CD58 with CD2 initiates cellular kinase signaling [28,29,30,31,32,33,34,35,36,37]. Previous studies have described the involvement of soluble factor/s in the inhibition of lymphocyte proliferation in peripheral blood mononuclear cells (PBMCs) infected with ASFV or incubated with cell extracts/supernatants free of virus [17,21] and have defined an immunomodulatory role of CD2v in inhibition of mitogen-induced proliferation of lymphocytes in vitro [21]. We hypothesized that soluble CD2v could be mimicking host CD2 by interacting with CD58, thus leading to induction of kinase signaling, and other downstream cellular events.

Here, we show ASFV CD2v induces NF-κB-mediated IFN expression through interaction with CD58. Treatment of swine PBMCs with purified CD2v leads to IFN-β induction, NF-κB-p65 nuclear translocation, and apoptosis thus providing a potential mechanism for CD2v-induced lymphocyte apoptosis. We propose that CD2v may be a significant viral factor in the bystander lymphocyte depletion observed during ASFV infection in pigs.

## 2. Materials and Methods

### 2.1. Cells

Porcine kidney cells (PK15) and monkey kidney cells (Vero) were obtained from the American Type Culture Collection (ATCC) and maintained at 37 °C with 5% CO_2_ in minimal essential medium (MEM) supplemented with 10% fetal bovine serum (FBS) (Atlanta Biologicals, Flowery Branch, GA, USA), 2 mM L-glutamine, gentamicin (50 μg/mL), penicillin (100 IU/mL) and streptomycin (100 μg/mL). Human embryonic kidney (HEK 293T) cells were maintained in the Dulbecco’s modified essential medium (DMEM) supplemented as above.

Swine PBMCs were obtained from swine whole blood through density gradient centrifugation using Sepmate15 (STEMCELL technologies, Vancouver, BC, Canada) and lymphocyte separation media (Corning, NY, USA) and frozen in freezing media (50% RPMI-1640, 40% FBS and 10% DMSO) as described elsewhere [38,39]. Swine PBMCs were maintained at 37 °C with 5% CO_2_ in RPMI 1640 medium (Corning, NY, USA) supplemented with 10% FBS, 2 mM L-glutamine, gentamicin (50 μg/mL), penicillin (100 IU/mL), streptomycin (100 μg/mL) and sodium pyruvate (1 mM).

Primary swine macrophage cells were prepared from swine whole blood as described previously [40]. Swine PBMCs were isolated as described above and examined at 24 h to confirm adherence to the substrate. Adherent primary macrophages were maintained at 37 °C with 5% CO_2_ in RPMI 1640 medium (Corning, NY, USA) supplemented with 20% fetal bovine serum (FBS) (Atlanta Biologicals, Flowery Branch, GA, USA), 30% L929 media, 2 mM L-glutamine, gentamicin (50 μg/mL), penicillin (100 IU/mL) and streptomycin (100 μg/mL). Primary macrophages were detached from cell culture plates using 10 mM EDTA in phosphate-buffered saline (PBS) and plated for experiments.

### 2.2. Plasmids and Transfection

The ASFV Congo strain CD2v gene was synthesized, cloned into pUC57 (Genscript, Piscataway, NJ, USA) and amplified with primers CD2v-Fw (*EcoRI*) (5′-TAAGGCCTCTGAATTCGCCACCATGATAATTAACTTATTTTTTTAATATG-3′) and CD2v-Rv (*KpnI*) (5′-CAGAATTCGCGGTACCAATAATTCTATCT ACATGAATAAGCG-3′). The virulent Congo K-49 is a HAI serogroup 2 virus isolated in Congo in 1949 and present in the Federal Research Center for Virology and Microbiology (FRCVM) ASFV strain repository [23,41]. The amplified full length *CD2v* gene was cloned into *EcoRI* and *KpnI* sites of pCMV-HA vector (Clontech, Mountain View, CA, USA) to produce pCD2v-HA, which express C-terminally HA-tagged CD2v protein (CD2v-HA). To enhance translation efficiency, a Kozak sequence (GCCACC) was placed in front of the *CD2v* gene. The final construct was sequenced to confirm sequence integrity and fidelity.

To construct expression plasmids pORFV120-Flag and pORFV113-Flag, ORFV120 and ORFV113 coding sequences were PCR-amplified from the orf virus strain OV-IA82 genome and cloned into the p3xFlag-CMV-10 vector (pFlag) (Clontech, Mountain View, CA, USA).

For transfection of cells, Lipofectamine 2000 (Invitrogen, Carlsbad, CA, USA) and plasmid DNA were separately diluted in the Opti-MEM medium (Gibco, MA, USA) and incubated for 5 min. Diluted DNA and lipofectamine 2000 were mixed (1:1 ratio) and incubated for 20 min. Finally, the DNA–lipid complex was added to cells for a 5 h incubation then, the Opti-MEM medium was replaced with 10% complete growth media.

### 2.3. Hemadsorption Assay

PK15 cells grown in 6-well plates were mock transfected or transfected with plasmid pCD2v-HA. At 24 h post-transfection (pt), culture media was removed and rinsed two times with PBS. Transfected cultures were incubated with PBS-washed 1% swine RBC overnight and rosette formation was scored with a microscope (×100).

### 2.4. CD2v Purification and Quantification

For CD2v protein purification, 293T cells were transfected with pCD2v-HA or pEmpty-HA control vector for 30 h. Cell lysates were harvested using the mammalian protein extraction reagent (MPER) (Thermo Scientific, Waltham, MA, USA) and incubated with anti-HA resin overnight using spin columns (Thermo Scientific, Waltham, MA, USA). Purified CD2v or purified control were eluted using HA peptides (1 mg/mL) (Thermo Scientific, Waltham, MA, USA) in Tris-buffer saline (TBS; Corning, NY, USA).

Quantification of purified CD2v was performed based on comparative densitometric analysis. Purified CD2v and purified control samples and bovine serum albumin (BSA) samples of known concentrations were run by SDS-PAGE, the gel was stained with Coomassie and the bands imaged with FluorChem R (Protein Simple, San Jose, CA, USA). Densitometric analysis was performed using ImageJ software and a standard curve was drawn. The concentration of purified CD2v obtained was approximately 3.5 ng/μL and 100 μL of CD2v preparation was used in individual experiments.

### 2.5. Monoclonal Antibodies against CD2v

PK15 cells transfected with pCD2v-HA were lysed at 30 h pt and lysates incubated overnight with the anti-HA antibody (Cell Signaling Technology, Danvers, MA, USA) at 4 °C, and immunoprecipitated using 50 μL of protein G agarose bead slurry (Millipore, Burlington, MA, USA). Monoclonal antibodies recognizing CD2v-HA were generated by immunizing BALB/c mice with immunoprecipitated CD2v-HA on agarose beads. To generate hybridomas, splenocytes from immunized mice were harvested and processed following the manufacturer’s protocols (STEMCELL technologies/ClonalCell-HY Hybridoma Cloning Kit, Vancouver, BC, Canada). Clones were screened for reactivity against CD2v-HA by immunofluorescence assay (IFA) using CD2v-HA-transfected PK15 cultures.

### 2.6. Western Blot

Fifty micrograms of whole cell protein extracts or 50 μL of the cleared culture supernatant were resolved by SDS-PAGE, blotted to nitrocellulose or PVDF membranes and probed with primary antibody against HA (2367; Cell Signaling Technology, Danvers, MA, USA), caspase-3 (9662S; Cell Signaling Technology, Danvers, MA, USA), PARP1 (sc-53643; Santa Cruz, CA, USA) or glyceraldehyde-3-phosphate dehydrogenase (GAPDH) (sc-25778; Santa Cruz, CA, USA). The blots were developed with appropriate HRP-conjugated secondary antibodies and chemiluminescent reagents (Thermo Scientific, Waltham, MA, USA) and imaged with FluorChem R (Protein Simple, San Jose, CA, USA). Densitometric analysis was performed using ImageJ software and all readings were normalized to GAPDH values.

### 2.7. Immunoprecipitation

293T cells or primary swine macrophages were transfected with pCD2v-HA and supernatants (at 24 h pt) and cell lysates (30 h pt) were collected in MPER lysis buffer (Thermo Scientific, Waltham, MA, USA). Supernatants and lysates were incubated overnight with the anti-CD2v monoclonal antibody mix or anti-HA antibody (3724; Cell Signaling Technology, Danvers, MA, USA). Pull down products were obtained by eluting in Laemmli buffer (Bio-Rad, Hercules, CA, USA) and analyzed by Western blot using the anti-HA antibody (2367; Cell Signaling Technology, Danvers, MA, USA).

### 2.8. CD58 siRNA Knockdown

To investigate involvement of CD58-CD2v interaction in CD2v-mediated IFN-β induction, siRNA knockdown experiments were performed using CD58 sense (S) (CUUCCAGAGCCAGAACUAU) and antisense (AS) (AUAGUUCUGGCUCUG GAAG) siRNA duplex (Sigma Aldrich, MA, USA). PK15 cultures were transfected with CD58 siRNA (15 nM) and mission siRNA transfection reagent (Sigma Aldrich, MA, USA) following the manufacturer’s protocol and transfected 24 h later with pCD2v-HA or control pEmpty-HA for 6 h. CD58 knockdown was assessed by comparing transcript levels between cultures transfected with one MISSION siRNA Universal Negative control (Sigma Aldrich, MA, USA) and CD58 siRNA-transfected cultures using RT-PCR. SYBR primers for swine CD58 were sCD58 FW 5′-ACTTAAACACTGGGTCGGGC-3′ and sCD58 RV 5′-AAGCTGCAAGGATCAGGCAT-3′.

### 2.9. Real-Time PCR

Interferon-β (IFN-β) and interferon stimulated gene (ISG) transcription were assessed in PK15 cells or primary swine macrophages transfected with pCD2v-HA or control plasmids and in swine PBMCs or primary swine macrophages treated with purified CD2v or purified control. Total RNA was harvested at various times post transfection/treatment with the RNA-extraction kit (Zymo, Irvine, CA, USA) and reverse transcribed with MLV-RT (Invitrogen, Carlsbad, CA, USA) as previously described [42]. IFN-β and ISGs mRNAs were quantified using ABI and the QuantStudio-3 Real time PCR system, Power SYBR Green PCR Master Mix (Applied Biosystems, Foster City, CA, USA) and primers sIFNβFw (5′-AGTGCATCCTCCAAATCGCT-3′) and sIFNβRv (5′-GCTCATGGAAAGAGCT GTGGT-3′) for IFN-β mRNA and sMX1Fw (5′-GGCGTGGGAATCAGTCATG-3′), sMX1Rv (5′-AGGAAGGTCTATGAGGGTCAGATCT-3′), sOAS1Fw (5′-GAGCTGCAGCGAGACTT CCT-3′) and sOAS1Rv (5′-TGCTTGACAAGGCGGATGA-3′) for ISGs *MX1* and *OAS*. Fold changes were calculated by comparison to Empty-HA or purified control for each time point. Individual experiments were conducted with biological triplicates and at least three technical replicates.

### 2.10. NF-κB-p65 Nuclear Translocation Assay

NF-κB activation was assessed by NF-κB-p65 nuclear translocation assays in (1) PK15 cells transfected with pCD2v-HA or control plasmids pORFV120-Flag and pORFV113-Flag, (2) swine PBMCs treated with purified CD2v or purified control and (3) swine macrophage treated with purified CD2v or purified control. At various times pts or protein treatments, cells were fixed with 2–4% paraformaldehyde (PFA) for 15 min and permeabilized with 0.2% triton ×100 (10 min). PK15 cells were incubated overnight at 4 °C with a primary antibody against HA (2367S; Cell Signaling Technology, Danvers, MA, USA), control Flag (A00187; Genscript, Piscataway, NJ, USA) and total NF-κB-p65 (8242; Cell Signaling Technology, Danvers, MA, USA). Likewise, PBMCs and swine macrophages were incubated with an antibody against total NF-κB-p65. PK15 cells were washed with PBS and incubated with secondary antibodies goat anti-mouse Alexa fluor 488 (A11029; Thermo Scientific, Waltham, MA, USA) to detect CD2v, ORFV120 and ORFV113 and goat anti-rabbit Alexa fluor 594 (A11012; Thermo Scientific, Waltham, MA, USA) to detect total NF-κB-p65. For PBMCs and swine macrophages the secondary antibody used to detect total NF-κB-p65 was goat anti-rabbit Alexa fluor 488 (A11008; Thermo Scientific, Waltham, MA, USA). PBMCs were deposited on slides using the Shandon cytospin 2 centrifuge (1500 rpm, 1 min). Nuclei were counterstained with DAPI and images obtained with an A1 Nikon confocal microscope. The number of cells exhibiting nuclear NF-κB-p65 staining was determined in randomly selected fields and results were expressed as a percentage of NF-κB-p65 expressing cells. 

For inhibition of the NF-κB-p65 nuclear translocation experiment, PK15 cells were pretreated with the NF-κB inhibitor parthenolide (InvivoGen, San Diego, CA, USA) at a 1 μM final concentration or vehicle control (DMSO) for one hour and transfected with pCD2v-HA in the presence or absence of parthenolide (1 μM). At 3 h pt, cultures were fixed and treated as above.

The role of the CD2v–CD58 interaction in the induction of NF-κB-p65 nuclear translocation was examined by transfecting PK15 cells with CD58 siRNA or siRNA universal negative control and 24 h later with pCD2v-HA. Cultures were fixed 3 h later, permeabilized and processed for HA and total NF-κB-p65 staining as described above. The percentage of cells containing nuclear NF-κB-p65 in CD2v-expressing cells in CD58 knockdown cells was determined. 

To investigate the effect of the anti-CD2v monoclonal antibody mix on CD2v-induced NF-κB-p65 activation in swine PBMCs, purified CD2v or purified control was incubated overnight at 4 °C with anti-CD2v monoclonal antibodies or anti-ORFV086 antibody or anti-IgG mouse isotype antibody control. PBMCs grown in 96-well plates were treated with preincubated CD2v or CD2v control (not preincubated with CD2v monoclonal antibodies), for 1.5 h and processed as above for NF-κB-p65 nuclear translocation. Randomly selected fields were scored for the mean percentage of cells containing nuclear NF-κB-p65. 

### 2.11. Interferon Bioassay

To investigate whether CD2v induced IFN-β and ISGs expression results in an antiviral state, an IFN bioassay was used. PK15 cells were transfected with pCD2v-HA or control plasmids pEmpty-HA or pORFV120-Flag, infected at 12 h, 24 h and 30 h pt with reporter vesicular stomatitis virus expressing GFP (VSV^GFP^; 50 PFU/well), fixed with 4% PFA at 16 h post infection and examined by IFA.

Supernatant obtained from PK15 cultures transfected with pCD2v-HA or control plasmids pEmpty-HA, pORFV120-Flag or Poly I:C (+control) were serially diluted and used to treat PK15 cells. At 30 h post-treatment, cultures were infected with VSV^GFP^ (50 PFU/well) and virus replication was assessed by IFA at 16 h post-infection.

To assess the antiviral activity in swine PBMCs treated with CD2v, swine PBMCs were treated with purified CD2v or purified control for 24 h and supernatants collected. Serially diluted PBMCs supernatants were then used to treat PK15 cells. To assess the antiviral activity PK15 cells were infected with VSV^GFP^ (50 PFU/well) 24 h post treatment, and virus replication was examined 16 h post infection by IFA.

### 2.12. Flow Cytometry

PK15 cells were transfected with pCD2v-HA or control plasmids and then infected with VSV^GFP^ at 12 h, 24 h and 30 h pt. At 16 h post infection, cells were trypsinized, fixed with 2% PFA and washed with PBS. GFP mean fluorescence intensity (MFI) was measured using the Cytek Aurora flow cytometer (Cytek Biosciences, Fremont, CA, USA).

CD2V-mediated activation of the NF-κB pathway was further examined using the flow cytometry assay to detect phosphorylated pNF-κB-p65 (S536). PK15 cells were transfected with pCD2v-HA or pORFV113-Flag, fixed with 2% PFA at 3 h pt, permeabilized with 0.2% Triton-X100 and incubated with anti-HA (2367S; Cell Signaling Technology, Danvers, MA, USA), anti-Flag (A00187; Genscript, Piscataway, NJ, USA) or antiphosphorylated NF-κB-p65 (S536) (3033S; Cell Signaling Technology, Danvers, MA, USA) for 45 min on ice. Secondary antibodies were goat anti-rabbit Alexa fluor 488 (A11008; Thermo Scientific, Waltham, MA, USA) for pNF-kB (S536) and goat anti-mouse Alexa fluor 647 (A21236; Thermo Scientific, Waltham, MA, USA) for CD2v and ORFV113. pNF-κB-p65 (S536) mean fluorescence intensity (MFI) was examined in cells expressing CD2v or ORFV113.

### 2.13. Coimmunoprecipitation

To study the interaction between CD2v and CD58, PK15 cells were cotransfected with pEmpty-HA and pCD58-Flag or pCD2v-HA and pCD58-Flag. Whole cell extracts were prepared at 8 h pt using the RIPA lysis buffer (Thermo Scientific, Waltham, MA, USA). Reciprocal coimmunoprecipitations were performed using the active motif Co-IP kit (IP High buffer) (Active Motif, Carlsbad, CA, USA) and protease inhibitor cocktail (Sigma Aldrich, MA, USA) following the manufacturer’s protocol. Whole cell extracts were incubated with anti-HA (3724S; Cell Signaling Technology, Danvers, MA, USA) and anti-Flag (A00187; Genscript, Piscataway, NJ, USA) antibodies overnight at 4 °C and then incubated with prewashed protein G agarose bead slurry (50 μL) (Millipore, Burlington, MA, USA) at 4 °C for 2 h. Beads were washed four times with IP high buffer and bound proteins eluted in the Laemmli buffer. Whole cell protein extracts and immunoprecipitated products were examined by SDS-PAGE-Western blot with the appropriate antibodies.

Interaction of CD2v with endogenous human CD58 was evaluated in 293T cells transfected with pCD2v-HA. Reciprocal coimmunoprecipitation was performed using anti-HA (3724S; Cell Signaling Technology, MA, USA) and hu-CD58 (sc20009; Santa Cruz, CA, USA) antibodies following the manufacturer’s protocol (Active motif Co-IP kit, Carlsbad, CA, USA). Immunoprecipitated products were resolved by SDS-PAGE, blotted to PVDF membranes and probed with primary antibody against HA (3724S; Cell Signaling Technology, Danvers, MA, USA) and hu-CD58 (sc20009; Santa Cruz, CA, USA) antibodies. Blots were developed with appropriate HRP-conjugated secondary antibodies (7074; Cell Signaling Technology, 7076; Cell Signaling Technology) and imaged with FluorChem R.

### 2.14. CD2v–CD58 Colocalization Assay

PK15 cells were cotransfected with pCD2v-HA and swine pCD58-Flag, fixed with 4% PFA 24 h pt, permeabilized with 0.2% Tritron-X 100 and treated with anti-HA (3724S; Cell Signaling Technology, MA, USA) or anti-Flag (A00187; Genscript, Piscataway, NJ, USA) primary antibodies. Cells were then washed with PBS and incubated with goat anti-rabbit Alexa fluor 594 (A11012; Cell signaling Technology, Danvers, MA, USA) and goat anti-mouse Alexa fluor 488 (A11029; Cell Signaling Technology, Danvers, MA, USA) secondary antibodies for 1 h. Nuclei were stained with DAPI and images obtained using the A1 Nikon confocal microscope. Colocalization of CD2v with endogenous human CD58 was investigated in 293T cells. Cells were transfected with pCD2v-HA and IFA was performed using anti-HA (3724S; Cell Signaling Technology, Danvers, MA, USA) and anti-CD58 (sc20009; Santa Cruz, CA, USA) primary antibodies, and goat anti-rabbit Alexa fluor 594 (A11012; Cell Signaling Technology, MA, USA) and goat anti-mouse Alexa fluor 488 (A11029) secondary antibodies.

### 2.15. TUNEL Assay

To investigate the effect of soluble CD2v on swine PBMC apoptosis. (1) Swine PBMCs treated with purified CD2v, purified control or staurosporine and (2) swine macrophages treated with purified CD2v, purified control or staurosporine were fixed at 18 h post-treatment, permeabilized as described above and stained for TUNEL following the manufacturer’s protocol (C10617; Thermo Scientific, Waltham, MA, USA). PBMCs were deposited on slides using Shandon cytospin 2 centrifuge (1500 rpm, 1 min). Nuclei were counterstained with DAPI and images obtained with an A1 Nikon confocal microscope. The number of cell nuclei exhibiting TUNEL staining were counted in randomly selected fields. Results were expressed as a percentage of TUNEL positive cells. 

### 2.16. Statistics

All statistical analyses were performed using Student’s *t* test. Statistically significant differences were indicated as * *p* < 0.05; ** *p* < 0.01 and NS, not significant.

## 3. Results

### 3.1. ASFV CD2v Localizes in the Perinuclear Region, Cytoplasm and Cell Membrane of PK15 Cells and Is Present in the Culture Supernatant

CD2v is an ASFV structural transmembrane glycoprotein of 360–407 amino acids with a predicted molecular weight of approximately 42–46 kDa and that is expressed on the surface of ASFV-infected macrophages [18,19]. CD2v mediates hemadsorption of swine red blood cells (RBCs) [18,19] and, together with viral C-type lectin, has a role in serotype specificity as defined by hemadsorption inhibition (HAI) [43].

**Figure 1:**
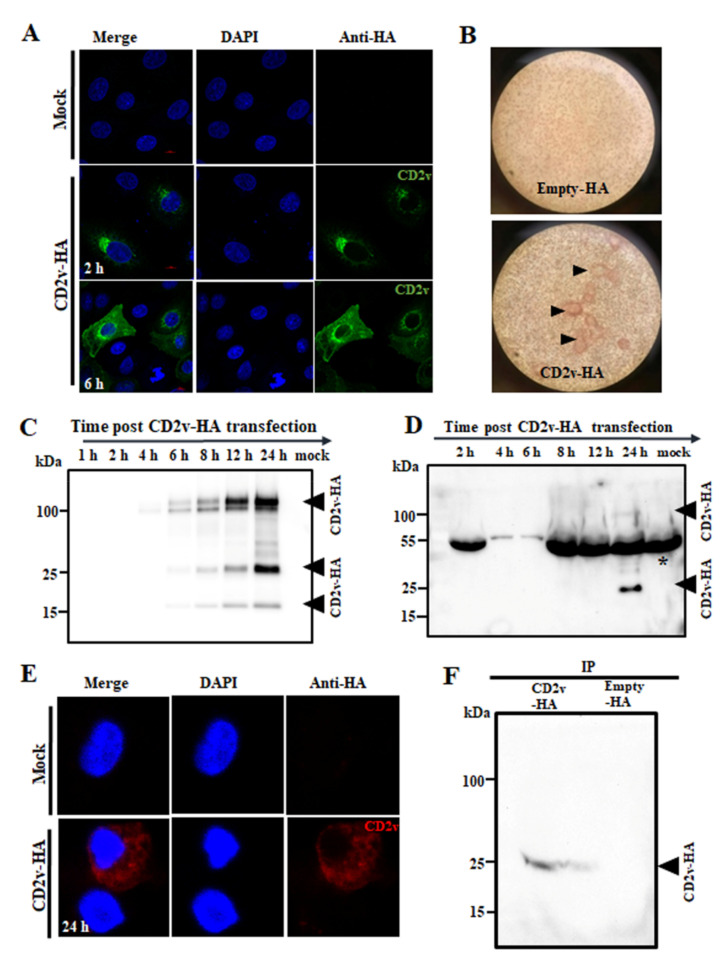
Subcellular localization and expression kinetics of ASFV CD2v. (**A**) PK15 cells were mock transfected or transfected with pCD2v-HA, fixed at various times pt, incubated with anti-HA primary antibody, sequentially stained with Alexa fluor 488-labeled secondary antibody and DAPI and examined by confocal microscopy. (**B**) CD2v-HA-expressing PK15 cells hemadsorbed swine red blood cells (RBCs). PK15 cells were transfected with plasmid pCD2v-HA for 24 h, incubated with swine RBCs overnight and examined using light microscopy (×100). CD2v-dependent rosetting is indicated by the arrowheads. (**C**) PK15 cells mock transfected or transfected with pCD2v-HA were harvested at the indicated times pt and total cell protein extracts were resolved by SDS-PAGE, blotted and incubated with antibodies against HA. (**D**) Detection of CD2v in the culture supernatant. PK15 cells were transfected as above and supernatants harvested at various times pt. Cleared supernatants (50 μL) were resolved by SDS-PAGE and analyzed by Western blot using antibodies against HA. * denotes the non-specific background band due to serum proteins in the supernatant. (**E**) Primary swine macrophages were mock transfected or transfected with a pCD2v-HA, fixed at 24 h pt, incubated with anti-HA primary antibody, sequentially stained with Alexa fluor 594-labeled secondary antibody and DAPI and examined by confocal microscopy. (**F**) Detection of CD2v in the culture supernatant of primary swine macrophages transiently expressing CD2v. Cells were transfected as above, incubated for 24 h and supernatants were immunoprecipitated with anti-HA antibody, resolved in SDS-PAGE and probed with the anti-HA antibody. Results for (**A**–**F**) are representative of two independent experiments.

To examine the subcellular localization and expression kinetics of CD2v, PK15 cells were mock transfected or transfected with pCMV plasmid expressing C-terminally HA-tagged CD2v (CD2v-HA) and examined at various times post-transfection (pt) by confocal microscopy. CD2v was observed adjacent to the nucleus at 2 h pt and in the cell membrane, perinuclear area and cytoplasmic vesicles at later times (Figure 1A). To confirm that CD2v expressed by PK15 cells is membrane localized and capable of mediating hemadsorption, PK15 cells were transfected with pEmpty-HA (control plasmid) or pCD2v-HA for 24 h, incubated with swine red blood cells (RBCs) overnight and scored for rosette formation. PK15 transfected with pCD2v-HA but not with control plasmid hemadsorbed swine RBCs as evidenced by rosette formation (Figure 1B, arrowheads).

The expression kinetics of CD2v was assessed by Western blot after transfection of PK15 cells with pCD2v-HA. Two major protein species of approximately 100 kDa and 25 kDa and a less-abundant 15 kDa species were detected at 6 h pt, with increasing protein levels observed at later time points (Figure 1C). A similar expression pattern was observed in 293T cells and Vero cells at 24 h pt (Figure S1A,B). The observed molecular weight of the full length protein was approximately 58 kDa higher than that predicted from the primary sequence. A major band of 42 kDa and a weaker band of 15 kDa were detected when CD2v was expressed in the presence of tunicamycin, an inhibitor of N-linked glycosylation, confirming that the protein is heavily modified through N-linked glycosylation (Figure S1C). The absence of the 25 kDa species in the presence of tunicamycin suggests the 25 kDa protein product might result from processing of the full length protein in the endoplasmic reticulum. It has been shown that CD2v is cleaved in the endoplasmic reticulum or Golgi compartments of virus-infected cells [20]. A faint 100 kDa and a predominant 25 kDa CD2v band were detected in the culture supernatant of PK15 cells 24 h post transfection with pCD2v-HA, indicating that CD2v is present in the culture supernatant (Figure 1D).

The subcellular localization of CD2v in primary swine macrophages was examined after mock transfection or transfection with pCD2v-HA at 24 h pt by confocal microscopy. Consistent with results in PK15 cells, CD2v was observed in the perinuclear area, cytoplasm and cell membrane of macrophages (Figure 1E). CD2v expression in primary swine macrophages was assessed by Western blot (WB) after transfection with pCD2v-HA. CD2v species of 75–100 kDa were detected in cell lysates at 24 h pt (Figure S1D). Although not evident in the WB, the 25 kDa CD2v band was detected by immunoprecipitation (IP) of the culture supernatant (Figure 1F).

These results are in agreement with previous studies on CD2v expression in ASFV-infected cells [18,20,44,45]. Notably, the study by Ruiz-Gonzalvo identified a soluble hemagglutinin in the media of ASFV infected macrophage cell cultures [45]. This soluble hemagglutinin may represent the soluble CD2v described here.

### 3.2. Expression of ASFV CD2v Induces IFN-β and ISG Transcription in PK15 Cells and Swine Macrophages

**Figure 2:**
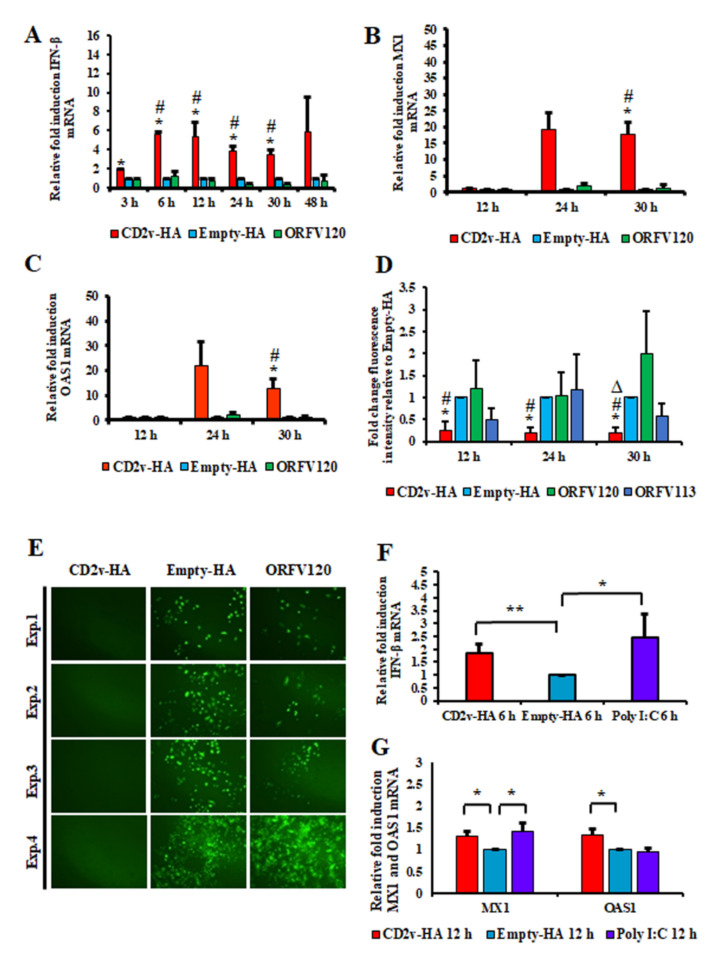
Induction of IFN-β, ISGs and the antiviral state in CD2v-expressing cells. PK15 cells were transfected with plasmids pCD2v-HA or controls pEmpty-HA and pORFV120-Flag (an unrelated viral transmembrane protein control) and transcription of IFN- β and selected ISGs was assessed by real-time PCR. (**A**) Shown are fold changes of IFN-β levels in cells transfected with pCD2v-HA relative to pEmpty-HA and pORFV120-Flag at various times pt. Results are the average mRNA levels from three independent experiments with *p*-values relative to controls < 0.05 at 3–30 h times pt. (**B**,**C**) Shown are fold changes of ISG mRNA levels at 12 h, 24 h and 30 h pt. Results are the average mRNA levels from three independent experiments. *p*-values relative to Empty-HA and ORFV120 at 30 h pt were 0.02 and 0.018 for *MX1* (**B**) and 0.04 and 0.037 for *OAS1* (**C**). * and # denote statistical significance compared to Empty-HA and ORFV120, respectively. (**D**,**E**) PK15 cells were transfected with pCD2v-HA, pEmpty-HA or control plasmids (pORFV120-Flag or pORFV113-Flag). At 12 h, 24 h or 30 h pt, cultures were infected with vesicular stomatitis virus expressing GFP (VSV^GFP^, 50 PFU/well). (**D**) Mean GFP fluorescence measured by flow cytometry at 16 h post infection. Results are the mean values of four independent experiments. *p*-values relative to transfection with control plasmids were < 0.05 at all times examined. *, # and ∆ denote statistical significance compared to Empty-HA, ORFV120 and ORFV113, respectively. (**E**) Fluorescence microscopy images taken at 16 h post infection with VSV^GFP^. Note decreased VSV replication in cells transfected with pCD2v-HA relative to controls. Results are representative of four independent experiments. Exp. denotes the experimental replicates. (**F**) Fold changes of IFN-β levels in swine macrophages transfected with pCD2v-HA relative to pEmpty-HA at 6 h pt. Results are the average from five independent experiments. *p*-value relative to Empty-HA was 0.008 for CD2v-HA. (**G**) Fold changes of ISG mRNA levels at 12 h pt. Results are the average from four independent experiments. *p*-values for CD2v-HA relative to Empty-HA were 0.014 for *MX1* and 0.03 for OAS1, respectively (*, *p* < 0.05; **, *p* < 0.01).

Preliminary RNA-Seq experiments were conducted to examine the effect of soluble CD2v on cellular gene transcription. PK15 cells were incubated with a CD2v-containing supernatant (1:2 dilution) or supernatant from cells transfected with pEmpty-HA (control plasmid) and total RNA was collected at 1 h, 2 h and 3 h post-treatment. RNA-Seq analysis showed upregulation of several interferon-stimulated genes (ISGs), including *MX1*, *OAS1* and *IRF9* at 2 h post treatment with a further increase at 3 h, suggesting a potential role of CD2v in IFN-β signaling. To assess the effect of CD2v expression on IFN-β and ISG transcription, PK15 cells were transfected with pCD2v-HA or control plasmids pEmpty-HA and pORFV120-Flag, the latter encoding for Orf virus protein ORFV120 and IFN-β transcription was assessed by RT-PCR. Compared to controls, cells transfected with pCD2v-HA plasmid showed significant upregulation of IFN-β (5.6-fold) as early as 6 h pt with similar upregulation observed at all subsequent time points sampled (Figure 2A). Consistent with upregulation of IFN-β, significant increases of ISGs *MX1* (17.7-fold) and *OAS1* (12.8-fold) transcription was observed at 30 h pt with pCD2v-HA plasmid compared to controls (Figure 2B,C).

To investigate the functional significance of IFN-β and ISGs induction by CD2v, the antiviral state of cells was examined using an IFN bioassay. PK15 cells were transfected with pCD2v-HA or control plasmids (Empty-HA vector or plasmids expressing Orf virus proteins ORFV120 and ORFV113) and then infected at various times pt with reporter vesicular stomatitis virus expressing GFP (VSV^GFP^, 50 PFU/well). PK15 cells transfected with pCD2v-HA but not with control plasmids showed an inhibition of VSV^GFP^ replication as determined by both flow cytometry and fluorescent microscopy at 12 h, 24 h and 30 h pt (Figure 2D,E). Inhibition of VSV^GFP^ replication was also observed when PK15 cell cultures were treated with CD2v containing supernatants (up to 1:4 dilution) but not with control supernatants (Figure S1E). These data indicate that CD2v expression in PK15 cells leads to the induction of IFN-β, ISGs and an antiviral state.

To examine the effect of CD2v on IFN-β and ISG transcription on macrophages, primary swine macrophage cell cultures were transfected with pCD2v-HA or pEmpty-HA or Poly I:C and IFN-β transcription was assessed by RT-PCR. Compared to pEmpty-HA control, significant upregulation of IFN-β was observed in macrophages transfected with pCD2v-HA plasmid (1.8-fold) and the positive control Poly I:C (2.4-fold) at 6 h pt (Figure 2F). Consistent with upregulation of IFN-β, a significant increase of ISGs *MX1* (1.3-fold) and *OAS1* (1.3-fold) transcription was observed at 12 h pt with the pCD2v-HA plasmid compared to the pEmpty-HA control (Figure 2G).

### 3.3. Induction of IFN-β by ASFV CD2v Is Dependent on NF-κB Activation

**Figure 3:**
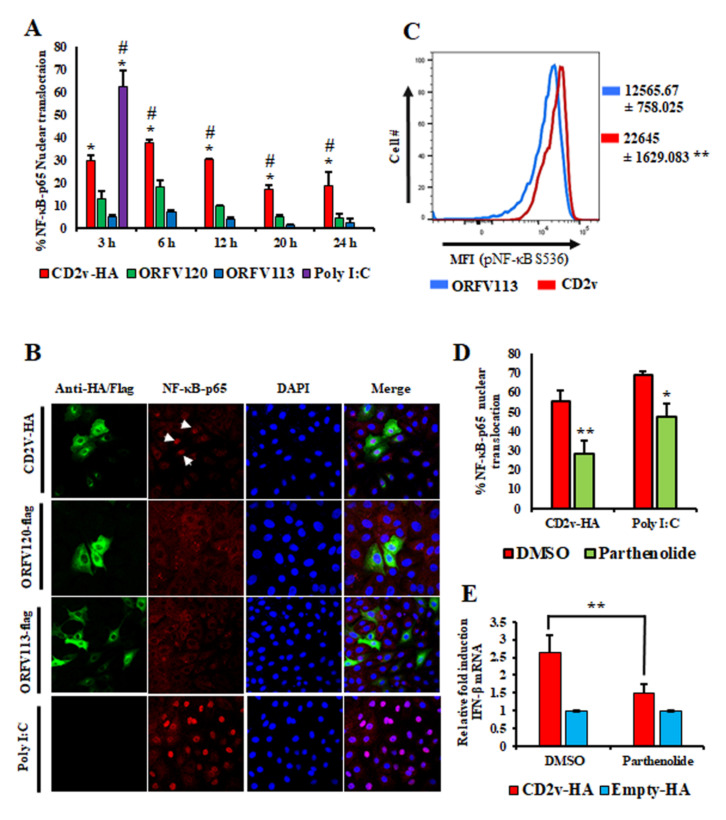
Expression of CD2v in PK15 cells induces IFN-β transcription in a NF-κB dependent manner. PK15 cells were transfected with pCD2v-HA or controls (pORFV120-Flag, pORFV113-Flag or Poly I:C), processed for immunofluorescence using primary antibodies against HA, Flag or NF-κB-p65 and secondary antibodies Alexa fluor 488 to detect CD2v, ORFV120 or ORFV113 and Alexa fluor 594 to detect NF-κB-p65 and counterstained with DAPI. Approximately 100 cells were counted/slide and results are shown as the mean values from three independent experiments. (**A**) Percentage of NF-κB-p65 nuclear translocation following the different treatments. *p*-values relative to controls ORFV120 and ORFV113 were 0.056 and 0.009 (3 h); 0.01 and 0.0006 (6 h); 0.019 and 0.02 (12 h); 0.005 and 0.004 (20 h); 0.016 and 0.009 (24 h), respectively. * and # denote statistical significance compared to ORFV120 and ORFV113, respectively. (**B**) Confocal microscopy images showing NF-κB-p65 nuclear translocation at 3 h pt (arrows). Green, CD2v or ORFV120 or ORFV113; Red, NF-κB-p65; Blue, DAPI. (**C**) Mean fluorescence intensity (MFI) of phosphorylated NF-κB (S536) measured by flow cytometry in CD2v and ORFV113 expressing cells at 3 h pt. Results are representative of three independent experiments. *p*-value relative to ORFV113 is 0.008. (**D**) PK15 cells pretreated with the NF-κB inhibitor parthenolide (1 μM) or DMSO (vehicle control) for one hour were transfected with pCD2v-HA or Poly I:C, fixed at 3 h pt and processed for immunofluorescence with antibodies against HA and NF-κB-p65. Approximately 100 cells were counted/slide and results are shown as mean values from three independent experiments (*p* = 0.001). (**E**) PK15 cells treated with parthenolide (1 μM) or DMSO for one hour were transfected with pCD2v-HA or pEmpty-HA in the presence or absence of parthenolide and IFN- β transcription assessed by RT-PCR at 6 h pt. Fold changes relative to Empty-HA and data are means from four independent experiments (*p* = 0.008). (*, *p* < 0.05; **, *p* < 0.01.)

NF-κB and IRF3 are two important transcription factors involved in IFN-β induction that translocate to the nucleus upon activation [46,47,48,49,50]. To assess whether CD2v expression affects NF-κB-p65 and IRF3 nuclear translocation, PK15 cells were transfected with pCD2v-HA or control plasmids (pORFV120-Flag and pORFV113-Flag) and examined by indirect immunofluorescence assay (IFA) at various times pt. Enhanced NF-κB-p65 nuclear translocation was observed in CD2v expressing cells at all times pt compared to controls (Figure 3A,B). In contrast, IRF3 nuclear translocation was not observed. To confirm the activation of the NF-κB pathway, PK15 cultures were transfected with pCD2v-HA or pORFV113-Flag (control) and the mean fluorescence intensity (MFI) of phosphorylated NF-κB (pNF-κB S536) was examined at 3 h pt by flow cytometry. Consistent with the nuclear translocation results, significantly increased MFI values (1.8-fold) were observed in CD2v-expressing cells compared to the control (Figure 3C). Inhibition of NF-κB activation with parthenolide, a NF-κB inhibitor, resulted in reduced nuclear translocation of NF-κB-p65 in CD2v-expressing cells (Figure 3D and Figure S1F).

To investigate the effect of NF-κB inhibition on CD2v-mediated IFN-β induction, PK15 cells were pretreated for one hour with parthenolide (1 μM) or DMSO (vehicle control), transfected with pCD2v-HA or pEmpty-HA (control) for 6 h in the presence of parthenolide (1 μM) or DMSO vehicle and assessed for IFN-β transcription by RT-PCR. Significant reduction in IFN-β transcription was observed in cells transiently expressing CD2v in the presence of parthenolide (1.5-fold) compared to cells expressing CD2v in the presence of the vehicle alone (2.6-fold) (Figure 3E). Together, the results above indicate that induction of IFN-β by ASFV CD2v was mediated by NF-κB activation.

### 3.4. CD2v–CD58 Interaction Mediates NF-κB Activation and IFN-β Transcription

**Figure 4:**
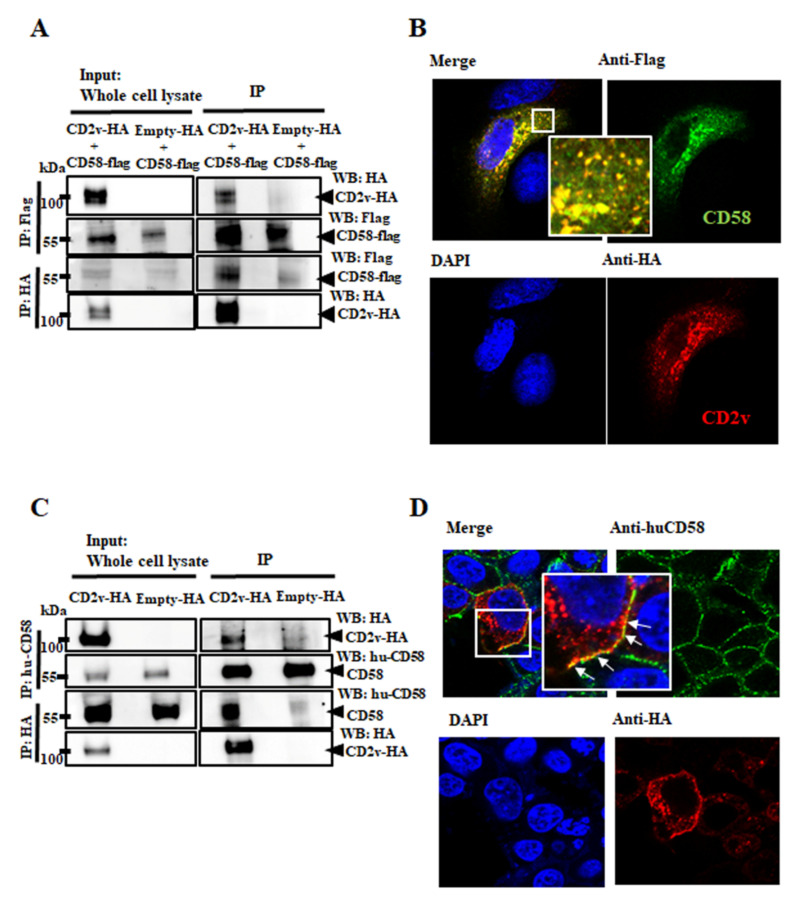
Interaction between CD2v and CD58. (A,B) Interaction between CD2v and porcine CD58. (**A**) PK15 cells were cotransfected with plasmids pCD58-Flag and pCD2v-HA or pCD58-Flag and pEmpty-HA (control) and harvested at 8 h pt. Whole cell lysates (left) and extracts immunoprecipitated with anti-Flag antibodies or anti-HA were examined by Western blotting with antibodies directed against proteins indicated on the right. (**B**) PK15 cells were cotransfected with pCD58-Flag and pCD2v-HA, fixed at 24 h pt, incubated with mouse anti-Flag and rabbit anti-HA primary antibodies, washed and incubated with secondary antibodies (Alexa-fluor 488-labeled anti-mouse and Alexa-fluor 594-labeled anti-rabbit). Cells were counterstained with DAPI and examined with the confocal microscope. Insets show magnified areas of the field. (**C**) 293T cells were transfected with pCD2v-HA or pEmpty-HA (control) and harvested at 8 h pt. Whole cell lysates (left) and extracts immunoprecipitated with mouse anti-huCD58 antibodies or anti-HA were examined by Western blotting with antibodies directed against proteins indicated on the right. (**D**) 293T cells were transfected with pCD2v-HA, fixed at 24 h pt, incubated with mouse anti-huCD58 and rabbit anti-HA primary antibodies, washed and incubated with Alexa-fluor 488-labeled anti-mouse and Alexa-fluor 594-labeled anti-rabbit secondary antibodies. Cells were counter stained with DAPI and examined with the confocal microscope. Insets show magnified areas of the field. Results for (**A**–**D**) are representative of three independent experiments.

CD2v contains all the domains present in cellular CD2 and some of the residues involved in binding to the CD58, the natural CD2 ligand [18,19]. To study the potential interaction between CD2v and CD58, PK15 cells were cotransfected with the pEmpty-HA vector or pCD2v-HA and porcine pCD58-Flag and cell lysates were prepared at 8 h pt for reciprocal coimmunoprecipitation with anti-Flag or anti-HA antibodies as described in Materials and Methods. Figure 4A shows that CD2v and porcine CD58 reciprocally coimmunoprecipitate.

To confirm CD2v–CD58 interaction, PK15 cells were cotransfected with pCD2v-HA and pCD58-Flag and localization of proteins was examined using confocal microscopy. A strong overlap of signals indicative of colocalization was observed (Figure 4B). Reciprocal coimmunoprecipitation and colocalization of CD2v with endogenous human CD58 was also observed at the plasma membrane of 293T cells transfected with pCD2v-HA (Figure 4C,D). Given that the host CD2–CD58 interaction activates downstream cellular kinases [28,29,30,31,32,33,34,35,36,37], we examined the involvement of the CD2v–CD58 interaction in CD2v-mediated NF-κB activation and IFN-β activation.

**Figure 5:**
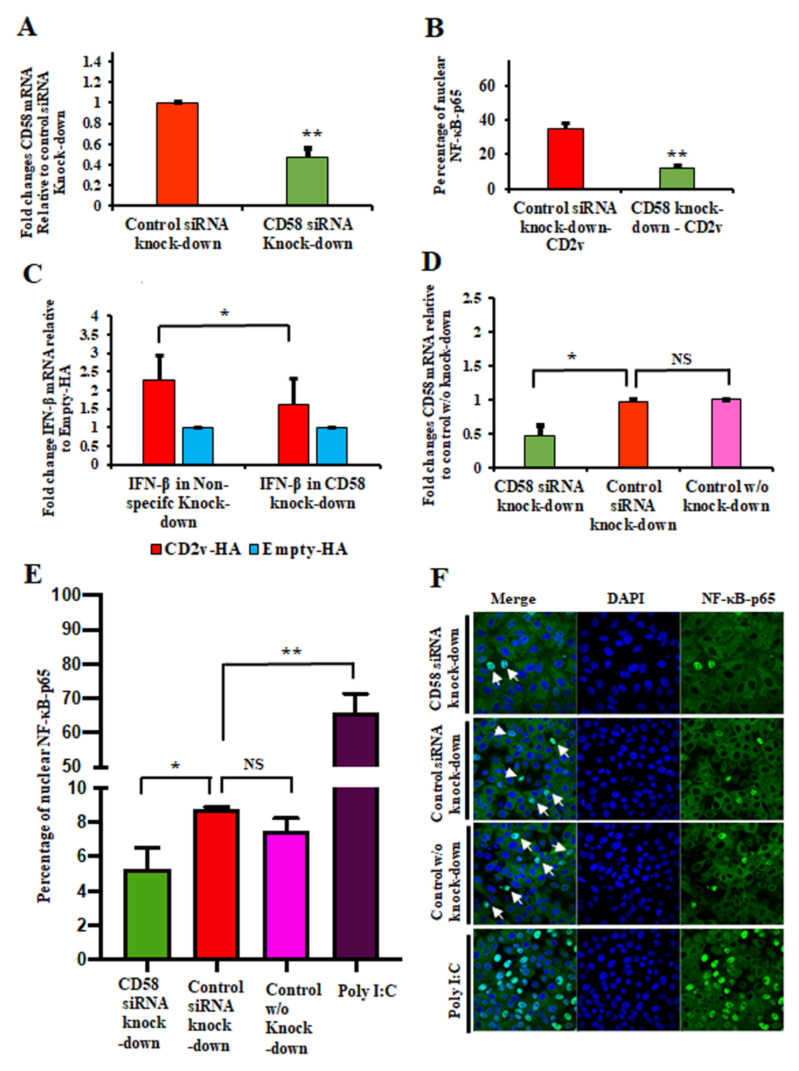
CD2v–CD58 interaction affects CD2v-mediated NF-kB-p65 nuclear translocation and IFN-β induction. (**A**) siRNA knockdown of CD58. PK15 cells were transfected with CD58 siRNA or siRNA universal negative control, total RNA was extracted at 24 h pt, cDNA prepared and CD58 transcription assessed by RT-PCR. Results are the mean of five independent experiments (*p* = 0.0002). (**B**) PK15 cells were sequentially transfected with CD58 siRNA or siRNA universal negative control and pCD2v-HA or pEmpty-HA, fixed at 3 h pt and processed for NF-κB-p65 detection by immunofluorescence. Approximately 100 cells were counted/slide and results are shown as mean values from three independent experiments (*p* = 0.002). (**C**) IFN-β induction. PK15 cells were sequentially transfected with CD58 siRNA or siRNA universal negative control and pCD2v-HA or pEmpty-HA. Total RNA was extracted at 6 h pt, cDNA prepared and IFN-β transcription assessed by RT-PCR. Fold changes are relative to Empty-HA and data are the mean mRNA levels from eight independent experiments (*p* = 0.025). (**D**) siRNA knockdown of CD58. PK15 cells were mock transfected or transfected with CD58 siRNA or siRNA universal negative control, total RNA was extracted at 24 h pt, cDNA prepared and CD58 transcription assessed by RT-PCR. Results are the mean of three independent experiments (*p* = 0.035). (**E**,**F**) NF-κB-p65 nuclear translocation following treatment with the purified CD2v protein. PK15 cells were sequentially mock transfected or transfected with CD58 siRNA or siRNA universal negative control and treated with purified CD2v protein, fixed at 2 h pt and processed for detection of NF-κB-p65 by immunofluorescence. Approximately 1000 cells were counted/slide and results are shown as mean values from three independent experiments (**E**). *p*-value for reduced CD58 transcript cultures relative to the siRNA negative control was 0.037. (**F**) Representative images of NF-κB-p65 nuclear translocation under conditions outlined above. Green, NF-κB-p65; Blue, DAPI. Arrows indicate nuclear NF-κB-p65. (*, *p* < 0.05 and **, *p* < 0.0.)

To evaluate the effect of CD58 downregulation on CD2v-mediated NF-κB activation, PK15 cells were transfected with siRNAs targeting porcine CD58 or control siRNA as described in Materials and Methods. CD58 transcript reduction of approximately 55% was routinely obtained in PK15 CD58 knockdown cells compared to the negative control (Figure 5A). Twenty-four hours following siRNA treatment, cells were transfected with pCD2v-HA for 3 h, and NF-κB-p65 nuclear translocation was assessed by confocal microscopy. A significant reduction in NF-κB-p65 nuclear translocation (60%) in PK15 CD58 knockdown cells was observed compared to control values (Figure 5B and Figure S2A). 

To investigate the involvement of CD2v–CD58 interaction in CD2v-mediated IFN-β induction, siRNA knockdown experiments and CD2v-HA/control transfections were performed as described above. Significant reduction of IFN-β transcription was observed 6 h pt with CD2v-HA in PK15 cells with reduced CD58 transcript levels (1.6-fold) compared to the control (2.3-fold) (Figure 5C).

To examine the functional interaction between soluble CD2v and CD58 expressed on PK15 cells in CD2v-mediated NF-κB activation, PK15 cells were transfected with siRNAs targeting porcine CD58 or control siRNA. CD58 transcript knockdown of approximately 52% was obtained in PK15 CD58 knockdown cells compared to the negative control (Figure 5D). Twenty-four hours post siRNA treatment, cells were treated with purified CD2v protein for 2 h and NF-κB-p65 nuclear translocation was assessed by confocal microscopy. Significant reduction in NF-κB-p65 nuclear translocation (39.5%) in purified CD2v treated cells was observed in PK15 CD58 knockdown cells compared to the control (Figure 5E,F).

### 3.5. Purified CD2v Induces NF-κB-p65 Nuclear Translocation and IFN-β Transcription in Swine PBMCs and Macrophage Cultures

Uncharacterized soluble factors released by ASFV-infected macrophages were shown to inhibit proliferation of swine lymphocytes in response to lectins [17] and CD2v was shown to be involved in the inhibition of mitogen-induced proliferation of bystander lymphocytes in virus-infected swine PBMC cultures [21]. This and the observation that CD2v is present in the supernatant of transfected cells (Figure 1D,F) and that cells treated with/or expressing CD2v (Figure 2) upregulate IFN-β and ISGs, leading us to hypothesize that (1) released CD2v induces IFN-β and ISGs expression in lymphocytes and macrophages and (2) induction involves the activation of the NF-κB-p65 signaling pathway. 

**Figure 6:**
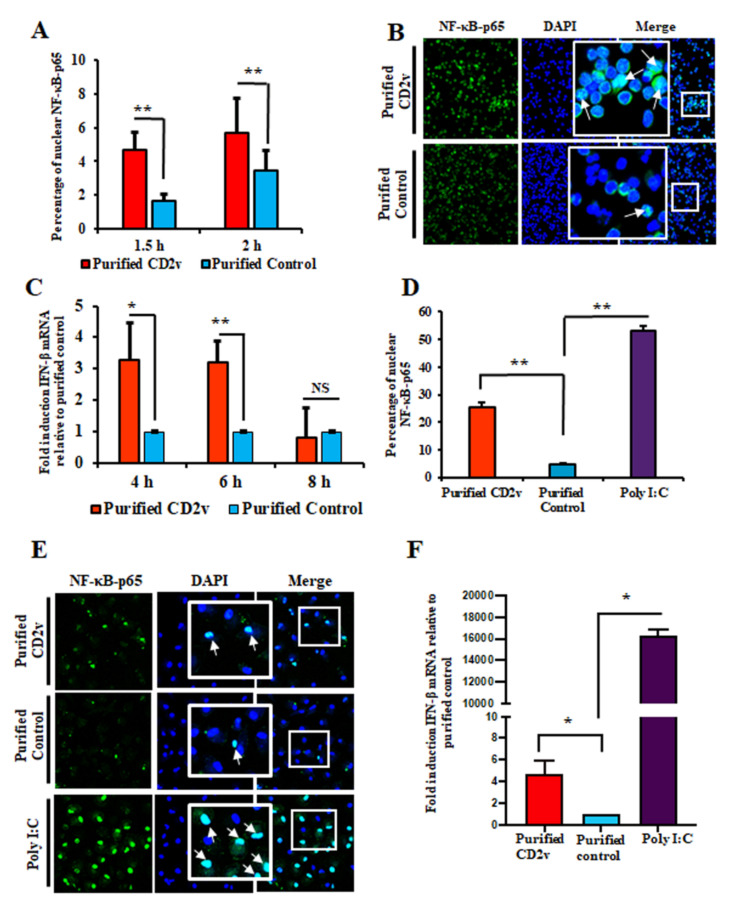
Purified CD2v protein induces NF-kB-p65 nuclear translocation and IFN-β transcription in swine PBMCs and macrophages. (**A**) NF-kB-p65 nuclear translocation in swine PBMCs. Swine PBMCs were treated with purified CD2v or purified control, fixed at 1.5 h and 2 h post treatment and assessed for NF-κB-p65 nuclear translocation by immunofluorescence as described in Materials and Methods. Approximately 2000 cells were counted/slide and results are shown as mean values from four independent experiments (1.5 h, *p* = 0.01; 2 h, *p =* 0.01). (**B**) Representative confocal images of NF-κB-p65 nuclear translocation. Green, NF-κB-p65; Blue, DAPI. Insets show magnified areas of the field. Arrows indicate nuclear NF-κB-p65. (**C**) IFN-β transcription. Cells were treated as in A, total RNA harvested at 4 h, 6 h and 8 h post treatment and IFN-β transcription assessed by RT-PCR. Fold changes are relative to purified control and data are the mean mRNA levels of seven independent experiments (4 h, *p* = 0.016; 6 h, *p* = 0.002). (**D**) NF-κB-p65 nuclear translocation in macrophages. Primary swine macrophages were treated with purified CD2v or purified control, fixed at 2 h post treatment and assessed for NF-κB-p65 nuclear translocation as in A. Approximately 300 cells were counted/slide and results are shown as the mean values from three independent experiments (for purified CD2v, *p* = 0.002). (**E**) Representative images of NF-κB-p65 nuclear translocation in macrophages. Green, NF-κB-p65; Blue, DAPI. Insets show magnified areas of the field. Arrows indicate nuclear NF-κB-p65. (**F**) CD2v-mediated induction of IFN-β in swine macrophages. Primary swine macrophages were treated with purified CD2v or purified control, the total RNA was harvested at 6 h post treatment and IFN-β transcription was assessed by RT-PCR. Fold changes are relative to the purified control and data are the mean mRNA levels of three independent experiments (for purified CD2v, *p* = 0.034). (*, *p* < 0.05 and **, *p* < 0.01).

To examine NF-κB-p65 nuclear translocation in swine lymphocytes, swine PBMCs were treated with purified CD2v or purified control for 1.5 or 2 h, processed for IFA and deposited onto glass slides with a cytospin. Confocal microscopy analysis showed enhanced NF-κB-p65 nuclear translocation in swine PBMCs treated with purified CD2v (2.8-fold at 1.5 h; 1.6-fold at 2 h) compared to the control treatment (Figure 6A,B).

To investigate whether IFN-β transcription in lymphocytes is affected by CD2v, swine PBMCs were treated as above and assessed for IFN-β transcription by RT-PCR at various times post-treatment. We found that IFN-β transcription was significantly induced in CD2v-treated PBMCs at 4 h (3.3-fold) and 6 h (3.2-fold) post treatment compared to the purified control (Figure 6C).

NF-κB-p65 nuclear translocation in macrophages was examined after the treatment of primary swine macrophages with purified CD2v or purified control for 2 h and processed for IFA. Confocal microscopy analysis showed enhanced NF-κB-p65 nuclear translocation in swine macrophages treated with purified CD2v at 2 h (5.4-fold) compared to the control treatment (Figure 6D,E). Additionally, macrophages treated with the CD2v protein exhibited increased IFN-β transcription (4.7-fold) at 6 h post treatment (Figure 6F).

### 3.6. Antibodies against ASFV CD2v Inhibit CD2v-Induced NF-κB Activation and IFN-β Transcription in Swine PBMC Cultures

Monoclonal antibodies against ASFV CD2v were generated and screened as described in Materials and Methods. Four anti-CD2v antibodies (A4, C4, C3 and F2) were pooled and used to examine their reactivity against CD2v. The antibody mixture detected the full length 100 kDa CD2v species in Western blot (Figure S2B). To confirm the reactivity of antibodies, lysates from 293T cells transfected with pCD2v-HA were incubated overnight with the anti-CD2v monoclonal antibodies. Immunoprecipitation products were assessed by Western blot using anti-HA antibodies. Both 100 kDa and 25 kDa CD2v species were observed in CD2v- but not in control-transfected cells (Figure S2C). 

**Figure 7:**
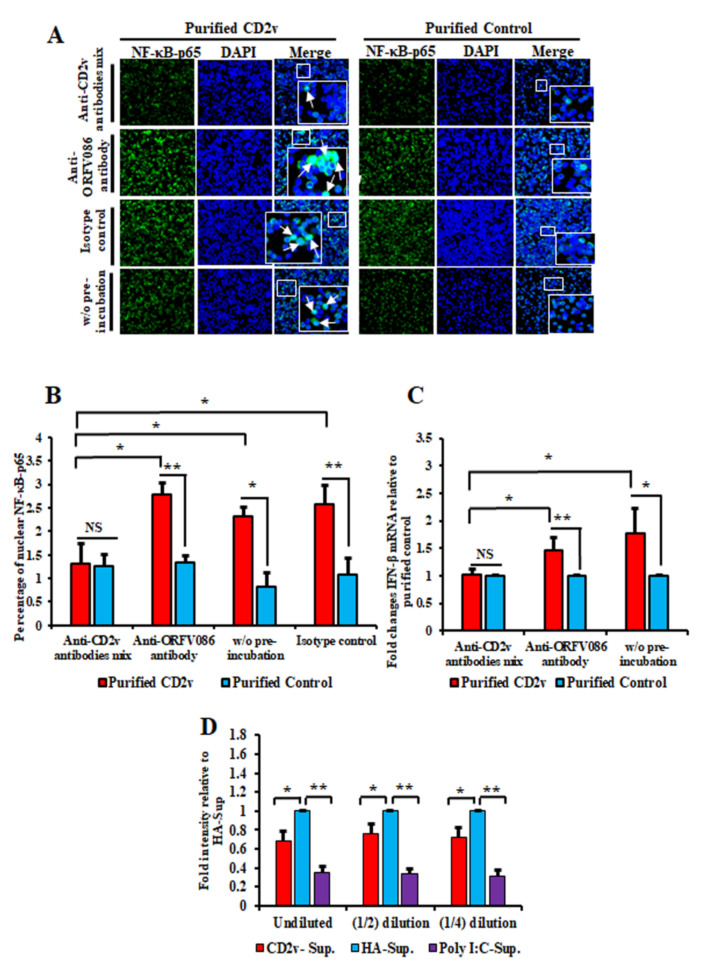
Monoclonal antibodies against ASFV CD2v inhibit CD2v-induced NF-κB activation and IFN-β transcription in swine PBMC cultures. (A,B) NF-κB-p65 nuclear translocation in PBMCs. Swine PBMCs were treated for 1.5 h with purified CD2v or purified control preincubated with the anti-CD2v monoclonal antibody mix, anti-ORFV086 antibody or isotype control antibody, and cells were processed for NF-κB-p65 staining as described in Materials and Methods. Control cells were treated with CD2v or the purified control that have not been preincubated with antibodies. (**A**) Representative confocal images of NF-κB-p65 nuclear translocation. Green, NF-κB-p65; Blue, DAPI. Arrows indicate nuclear NF-κB-p65. Insets show magnified areas of the field. (**B**) Approximately 2500 cells were counted/slide and results are shown as the mean values from three independent experiments (for % nuclear NF-κB-p65 for the anti-CD2v antibody mix relative to anti-ORFV086, *p* = 0.01; without preincubation, *p* = 0.03 and for the isotype control, *p* = 0.03). (**C**) IFN-β transcription in PBMCs. Swine PBMCs were treated as in A, total RNA harvested at 6 h pt and IFN-β transcription assessed by RT-PCR. Fold changes are relative to the purified control and data are mean values from five independent experiments. *p*-value for IFN-β fold induction for anti-CD2v antibody mix compared to anti-ORFV086 is 0.014 and without preincubation is 0.028. (**D**) Swine PBMCs were treated with the purified CD2v protein or purified control and supernatants collected 24 h post treatment. The supernatant collected from PK15 cells transfected with Poly I:C served as a positive control. PK15 grown in 96 well plates were treated with different dilutions of PBMCs supernatants for 24 h and subsequently infected with VSV^GFP^ (50 PFU/well) for 16 h. Intensity (mean gray value) was measured by ImageJ. Fold changes are relative to the purified control and data are the mean of four independent experiments. *p*-values relative to purified control for undiluted, 1:2 diluted and 1:4 diluted supernatants were 0.014, 0.033 and 0.017, respectively. (*, *p* < 0.05 and **, *p* < 0.01.)

To investigate the effect of the anti-CD2v antibodies on CD2v-induced NF-κB activation in swine PBMCs, purified CD2v or purified control were incubated overnight with the monoclonal antibody mix, control anti-ORFV086 monoclonal antibody or anti-IgG mouse isotype antibody control. Swine PBMCs were then treated with purified CD2v or purified control preincubated with the various antibodies and assessed for NF-κB-p65 nuclear translocation as above. As a control, purified CD2v or the purified control without preincubation with antibodies was used. Significant inhibition of NF-κB-p65 nuclear translocation (approximately 50% reduction) was observed in PBMCs treated with CD2v previously incubated with anti-CD2v monoclonal antibodies compared to controls (Figure 7A,B). This result show that anti-CD2v antibodies interfered with the ability of soluble CD2v to induce NF-κB-p65 nuclear translocation in swine PBMCs.

The effect of the anti-CD2v monoclonal antibodies on CD2v-induced IFN-β expression in swine PBMCs was investigated by preincubating purified CD2v or purified control with the antibodies described above, followed by treatment of PBMCs. As a control, purified CD2v or purified control without preincubation with antibodies was used to treat swine PBMCs. Total RNA was extracted 6 h post treatment and IFN-β transcription was assessed by RT-PCR. Significant inhibition in IFN-β transcription was observed when swine PBMCs were treated with purified CD2v preincubated with the anti-CD2v antibody mix (1-fold) as compared to purified CD2v preincubated with the anti-ORFV086 monoclonal antibody (1.5-fold) or without preincubation (1.8-fold) (Figure 7C). These results confirm a role for soluble CD2v in the induction of IFN-β transcription in swine PBMCs.

### 3.7. Supernatants from CD2v-Treated Swine PBMCs Exhibit Antiviral Activity

To determine whether the induction of IFN-β by CD2v is associated with antiviral activity in PBMCs, swine PBMCs were treated with purified CD2v or purified control for 24 h and the supernatants collected, diluted and used to treat fresh PK15 cells. Twenty-four hours after treatment the cells were infected with VSV^GFP^ and examined for virus replication at 16 h post infection by IFA. The supernatant collected from the PK15 cell culture transfected with Poly I:C was used as a positive control. Significant inhibition of VSV^GFP^ replication was observed in PK15 cells treated with the supernatant from CD2v treated PBMCs. We observed 32.1%, 24.4% and 28.1% inhibition with undiluted supernatant, 1:2 dilution and 1:4 dilution, respectively (Figure 7D and Figure S3A).

### 3.8. CD2v Induces Apoptosis in Swine PBMCs and Macrophage Cultures

ASF is characterized by severe destruction of lymphoid tissue and massive lymphocyte depletion due to apoptosis [7,8,9,51,52]. An explanation for this critical pathogenic event is lacking. ASFV replicates in cells of the monocyte lineage, most notably macrophages, but not in lymphocytes, thus apoptosis in bystander lymphocytes is most likely due to proteins or factors released by infected macrophages. Based on our data, we hypothesized that CD2v released by infected macrophages in lymphoid tissues induces IFN expression in bystander lymphocytes and macrophages leading to apoptosis. 

**Figure 8:**
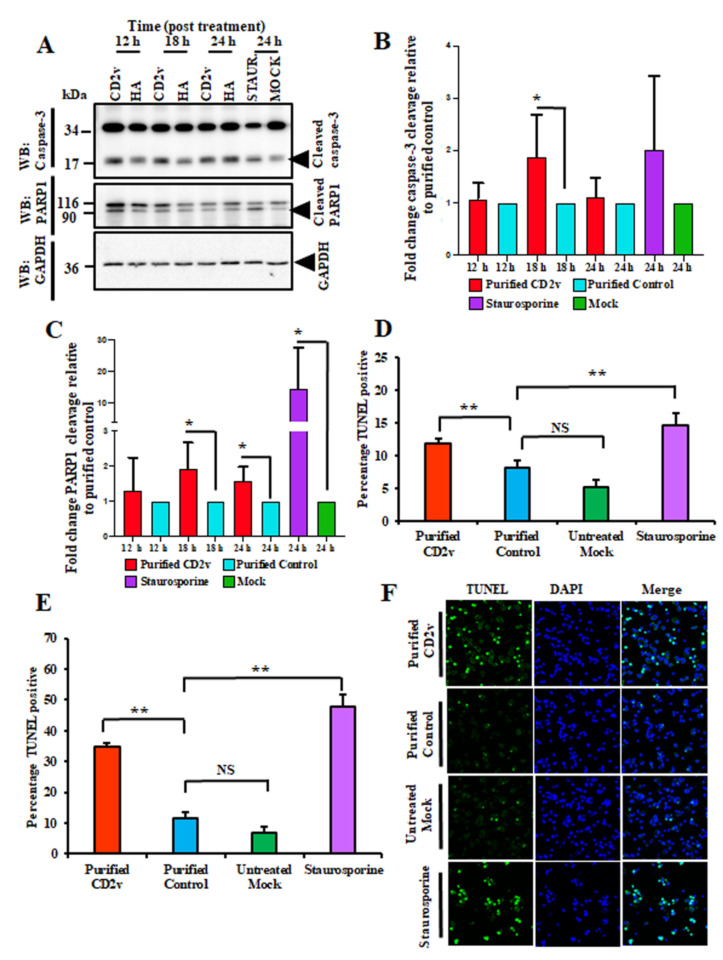
Purified CD2v protein induces apoptosis in swine PBMCs. (**A**) Caspase-3 and PARP1 cleavage. Swine PBMCs were treated with purified CD2v, purified control or staurosporine (positive control) and whole cell lysates were obtained at various times post treatment, resolved by SDS-PAGE, blotted and probed with antibodies against Caspase-3, PARP1 and GAPDH (loading control). (**B**) Densitometric analysis showing the fold change in caspase-3 activation relative to the purified control treatment. The caspase-3 results are the mean values of six independent experiments (18 h, *p* = 0.042). (**C**) Densitometric analysis showing fold changes in PARP1 cleavage relative to the purified control treatment. Results are the mean values of six independent experiments (18 h, *p* = 0.026; 24 h, *p* = 0.018). (**D**) TUNEL assay in lymphocytes. Swine PBMCs treated as in A were fixed at 18 h post treatment and processed for the TUNEL assay as described in Materials and Methods. Approximately 5000 lymphocytes (6–9 μm in diameter) were counted/slide and results are shown as mean values from three independent experiments. *p*-values for purified CD2v relative to the purified control and mock were 0.004 and 0.006, respectively. (**E**) TUNEL assay in macrophages. Swine macrophages (16–23 μm in diameter) treated as in (**A**) were fixed at 18 h post treatment and processed for the TUNEL assay as described in Materials and Methods. Approximately 1600 cells/slide were counted and results are mean values of three independent experiments. *p*-values for purified CD2v relative to the purified control and mock were 0.004 and 0.003, respectively. (**F**) Representative confocal images of the TUNEL assay under conditions outlined in (**E**). Green, TUNEL; Blue, DAPI. (*, *p* < 0.05 and **, *p* < 0.01.)

To investigate the effect of CD2v on PBMCs apoptosis, swine PBMC cultures were treated with purified CD2v, purified control or staurosporine (positive control) and caspase-3 and PARP1 cleavage assessed by WB at various times post treatment as described in the Materials and Methods. As shown in Figure 8A–C, treatment of swine PBMCs with purified CD2v led to significant induction of caspase-3 activation at 18 h post treatment (1.9-fold) and PARP1 cleavage at 18 h (1.9-fold) and 24 h (1.6-fold) post treatment compared to the purified control. 

Additionally, induction of apoptosis in swine PBMCs by the CD2v treatment was assessed by the TUNEL assay. Treatment of swine PBMCs with purified CD2v led to a significant increase in the percentage of TUNEL positive lymphocyte nuclei (1.5-fold) and TUNEL positive macrophages/monocytes nuclei (2.8-fold) at 18 h post treatment compared to the purified control (Figure 8D–F and Figure S3B). These results indicate that CD2v induced apoptosis in swine PBMCs and macrophages. 

## 4. Discussion 

A hallmark of acute ASF is the severe lymphoid tissue destruction and massive lymphocyte depletion in infected pigs, which occurs as a result of bystander lymphocyte apoptosis [4,8,52]. Since lymphocytes do not support ASFV replication, factors released or secreted by infected macrophages have been implicated in triggering lymphocyte apoptosis [7,8,9,10,11]. CD2v, an ASFV glycoprotein, has been shown to be involved in host immunomodulation, virulence and induction of protective immune responses [21,22,23]. We observed that treatment of swine PBMCs and macrophages with purified CD2v leads to significant NF-κB activation, IFN-β induction, (Figure 6A–F) and lymphocyte/macrophage apoptosis (Figure 8A–F and Figure S3B). These findings had important implications for ASFV pathogenesis.

Nearly all cell types produce type I IFNs when host pathogen recognition receptors (PRRs) bind different pathogen-associated molecular patterns (PAMPs) [53]. IFN signaling leads to transcriptional induction of interferon stimulated genes (ISGs), which mediate most antiviral, antiproliferative, proapoptotic and immunomodulatory functions of IFN [54]. The proapoptotic IFN-β function occurs through the activation of either the intrinsic or extrinsic apoptotic pathway and ISGs such as OAS proteins have been shown to play a role in the induction of apoptosis [55,56,57,58,59,60,61,62]. Here, AFSV CD2v induced IFN-β/ISGs transcription, including OAS1 transcription, suggesting a mechanism for apoptosis in swine PBMCs and macrophages.

IFN-β induction is mediated by two major groups of transcription factors, nuclear factor-kB (NF-κB) and IFN-regulatory factors (IRFs) [46,47,48,49,50]. Due to the central role of NF-κB in various antiviral responses, viruses target multiple steps of the NF-κB activation pathway, from PRR recognition to NF-κB mediated gene transcription [63]. Results here implicate CD2v activation of NF-κB, but not IRF3, in the induction of IFN-β (Figure 3A–E, Figure 6A–F and data not shown). IFN induction of NF-κB has been observed for other viruses [47,48,64,65].

The relationship between ASFV infection and the IFN system is complex. ASFV has evolved multiple strategies to modulate activation of the IFN and NF-κB signaling pathways [66,67,68,69]. The ASFV genome encodes several genes that function to interfere with NF-κB and IFN pathways in macrophages. For example, genes of the multi-gene family 360 and 505 (MGF360/MGF505) suppress IFN induction through yet unidentified mechanisms, while DP96, A238L and I239L use different strategies to inhibit NF-κB [66,67,68,69]. In vitro infection of porcine macrophages with low virulence ASFV strains led to enhanced and sustained IFN production compared to virulent strains [13,14,70,71,72,73]. In vivo acute ASFV infection of pigs with highly virulent virus strains is characterized by elevated levels of systemic IFN and cytokine (TNFα, IL-1α, IL-1β and IL-6) production [8,9,10,11,74,75], suggesting a potential role/s of IFNs in ASF pathogenesis. Our data suggest that CD2v may upregulate IFN levels in lymphoid tissues and this response may be involved in the elevated IFN levels observed during acute infection.

CD58/LFA-3 is the natural ligand for host CD2 protein and CD2–CD58 interaction initiates cellular kinase signaling [28,29,30,31,32,33,34]. CD2 has been shown to activate T cells through tyrosine kinase ZAP-70 and protein kinase C (PKC) [35,36,37]. CD2 costimulation was shown to trigger strong MEK/ERK1/2 phosphorylation and weak NF-κB phosphorylation in T cells [37]. There are two isoforms of CD58: type-I transmembrane and glycosylphosphatidylinositol (GPI)-anchored forms and both forms are associated with protein kinases [76,77]. CD58 signaling induces phosphorylation of PLCγ, SYK and BLINK and activates AKT and ERK transcription factors [78]. In addition, treatment of cells with antibodies against CD58 also activates CD58 signaling [79,80,81].

CD59 and CD48 are additional CD2 ligands, which binds to CD2 with low-affinity [82,83,84,85]. CD59 and CD58 binding regions in CD2 are overlapping but not completely identical whereas CD48 and CD58 both binds to the T11 region on CD2 [84,86]. Although the CD2–CD59 interaction alone was not sufficient to cause T-cell activation, it was shown to promote CD58-mediated T-cell activation and IL-production [87]. CD48 is a murine homolog of human CD58 [85,88,89]. CD58 is suspected to have originated by gene duplication from CD48, evolving into a major CD2 interactor, during mammalian evolution after divergence from the mouse [89]. Given that CD2 can interact with CD59 and CD48, we cannot exclude the possibility that ASFV CD2v also might interact with these molecules. However, we clearly show that swine CD58 interacts with CD2v and that the interaction results in NF-κB activation and IFN-β induction (Figure 4 and Figure 5). Since viral glycoproteins can also induce IFN-β through TLR-4/TLR-2 [90,91,92,93], involvement of these receptors in the results shown here cannot be formally excluded.

In ASFV-infected macrophages, CD2v is processed and thought to be released [20,45]. Here, we show that CD2v is present in the supernatant of CD2v-expressing cells (Figure 1D,F). The mechanism responsible for this is yet to be determined. The membrane-associated protein may be secreted or leave the cell by membrane blebbing or via exosomes. Notably, Yang et al. recently have shown that a CD2v C-terminal 88 amino acid fragment is able to enter cells when present in the culture supernatant [94]. However, the effect is lost on the removal of CD2v C-terminal repeat sequences ([KPCPPP]3) [94]. Interestingly, these sequences are conserved in ASFV isolates and present in all of the CD2v species (100 kDa, 25 kDa and 15 kDa) described here. Conceivably, this mechanism could be involved with the protein entry and/or egress from the cell. CD2v processing or interaction with the ER and trans-Golgi network AP-1 factor and actin-binding adaptor protein SH3P7 has been described and may also be involved in some manner [20,44,95].

Previous studies have described the involvement of soluble factor/s in the inhibition of lymphocyte proliferation in PBMCs infected with ASFV or incubated with cell extracts/supernatants free of virus [17,21] and have defined an immunomodulatory role of CD2v in the inhibition of mitogen-induced proliferation of lymphocytes [21]. CD2v may represent the soluble hemagglutinin previously described in supernatants of ASFV infected macrophages [45].

The ASFV CD2v protein has been implicated as a protective antigen (PA) for ASFV. CD2v gene orthologues are among the most divergent between genome sequences of ASFV isolates [1,96], providing antigens of potential significance in serogroup-specific immunity [97]. Although available data support a role for both humoral and cellular immune responses in protection, definitive immune correlates of protection are lacking [98]. However, the protection afforded by passive transfer of ASFV antibodies provides compelling evidence for antiviral antibodies in protective immunity [99,100,101]. Pigs immunized with CD2v developed hemadsorption inhibiting (HAI) and monocyte infection-inhibiting (M-II) antibodies that recognized a 75 kDa virion protein and they were partially protected from challenge with the homologous virulent virus strain [45,102]. Our observation that anti-CD2v antibodies inhibit CD2v-dependent NF-κB activation and IFN-β induction (Figure 7A–C) suggests that neutralization of CD2v by anti-CD2v antibodies may be an important antibody-mediated protective immune mechanism.

**Figure 9:**
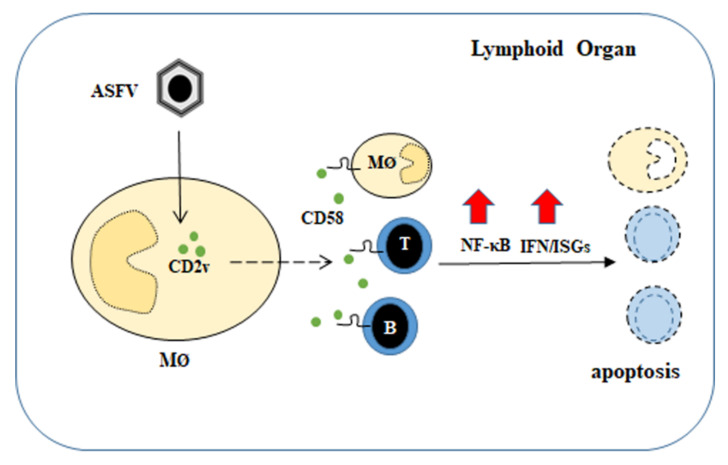
Proposed mechanism of action of ASFV CD2v. CD2v present on infected cell membranes and/or released from ASFV-infected macrophages interacts with surrounding lymphocytes and macrophages via CD58. This interaction promotes NF-κB activation and induction of IFN-β and ISGs, which lead to the apoptosis of lymphocytes and macrophages. A dashed arrow indicates that the mechanism responsible for CD2v presence in the supernatant is yet to be determined.

In conclusion, our data indicate a previously undescribed role for CD2v and suggests that protein is a contributing factor to the lymphoid tissue damage and lymphocyte depletion observed during acute ASFV infection (Figure 9).

## Data Availability

Data supporting the reported results are available in this article and in the Supplementary Materials.

## Supplementary Materials

The following are available online at https://www.mdpi.com/article/10.3390/v13081480/s1, Figure S1: (A,B) Expression of CD2v in 293T and Vero cells, (C) CD2v expression in presence of tunicamycin, (D) Expression of CD2v in primary swine macrophages, (E) Induction of antiviral state, (F) Representative confocal images of NF-κB-p65 nuclear translocation in PK15 cells under conditions outlined in Figure 3D, Figure S2: (A) Effect of CD58 knock-down on CD2v-induced NF-κB-p65 nuclear translocation, (B) Monoclonal antibodies generated against ASFV CD2v identifies CD2v in Western blots, (C) Immunoprecipitation with anti-CD2v monoclonal antibodies, Figure S3: (A) Antiviral activity of supernatants from CD2v -treated swine PBMCs, (B) Representative confocal images of TUNEL assay in swine PBMCs under conditions outlined in Figure 8D.
