# Supplementary material for: African Swine Fever Virus CD2v Protein Induces β-Interferon Expression and Apoptosis in Swine Peripheral Blood Mononuclear Cells
Source: Viruses. 2021 Jul 28;13(8):1480. doi: 10.3390/v13081480 (PMC8402892; doi:10.3390/v13081480)
Supplement: Supplementary file 1 [file viruses-13-01480-s001.zip › viruses-1304227-supplementary.pdf]

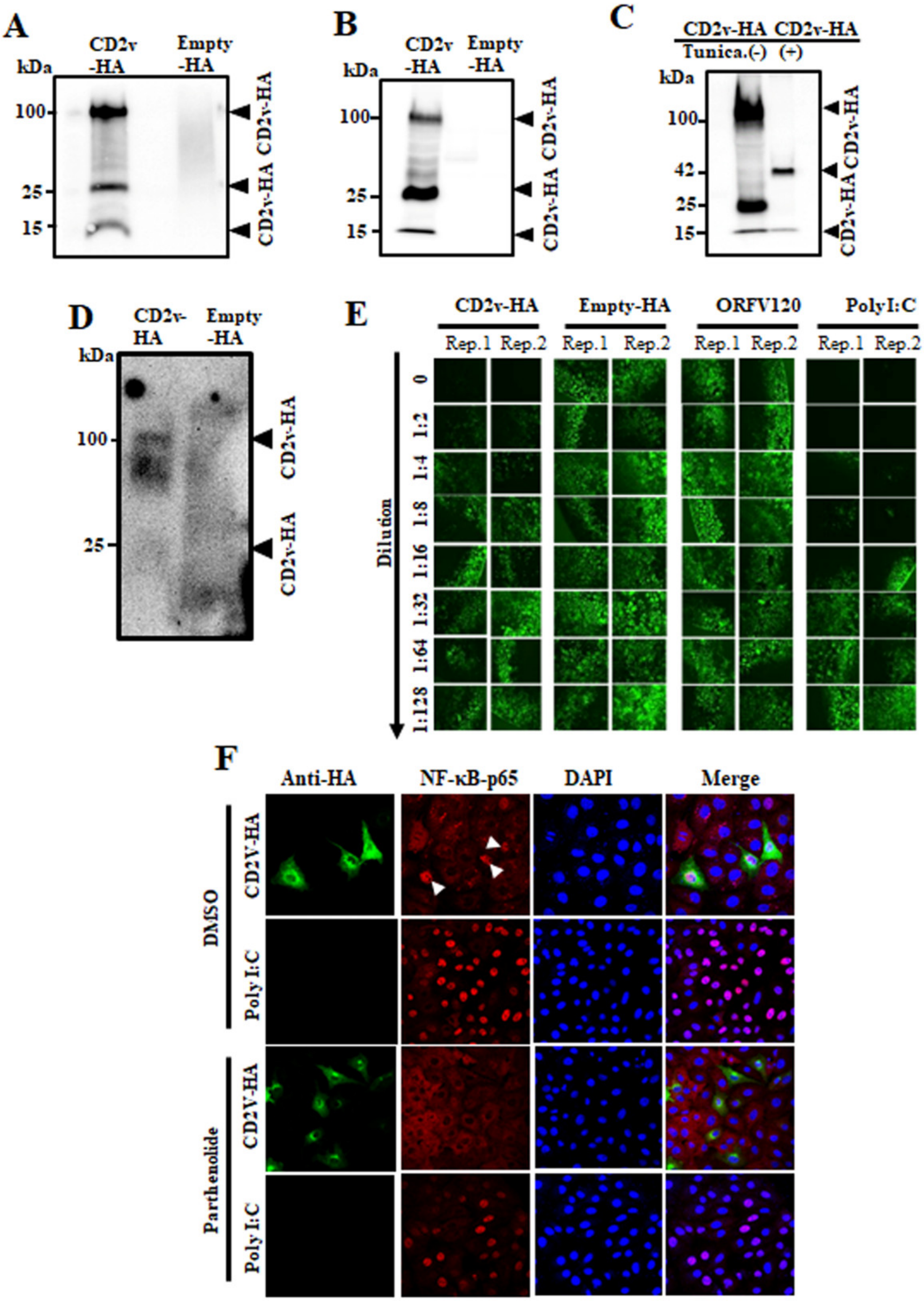

FIG S1

Figure S1. Expression of CD2v in 293T and Vero cells. 293T (**A**) and Vero cells (**B**) mock transfected or transfected with plasmid pCD2v-HA were harvested at 24 h post transfection.

Total cell protein extracts were resolved by SDS-PAGE, blotted and incubated with antibodies against HA. Results are representative of two independent experiments. (**C**) CD2v expression in presence of tunicamycin. PK15 cells transfected with pCD2v-HA. Five hours post-transfection, media was replaced with complete growth media containing 1μg/ml tunicamycin and incubated for 24 hr. Total cell extracts were resolved by SDS-PAGE, blotted and incubated with antibodies against HA. Results are representative of two independent experiments. (**D**) Primary swine macrophages, mock transfected or transfected with plasmid pCD2v-HA, were harvested at 24 h post transfection. Total cell protein extracts were resolved by SDS-PAGE, blotted and incubated with antibody against HA. (**E**) Induction of antiviral state. PK15 cells were treated with supernatants obtained from cultures transfected with pCD2v-HA, pEmpty-HA or pORFV120-Flag, or with poly I: C, and infected with VSV^{GFP} (50 PFU/well) 30 h post treatment. Shown are fluorescence microscopy images taken at 16 h post infection. Results are representative of two independent experiments. Rep., denotes technical replicates. (**F**) PK15 cells pretreated with the NF-κB inhibitor parthenolide (1 μM) or DMSO (vehicle control) for one hour, were transfected with pCD2v-HA, fixed at 3 h post transfection and processed for immunofluorescence with antibodies against HA and NF-κB-p65. Representative confocal images of cells treated as above. Green, CD2v; Red, NF-κB-p65; Blue, DAPI. Arrows indicate nuclear NF-κB-p65. Results are representative of three independent experiments.

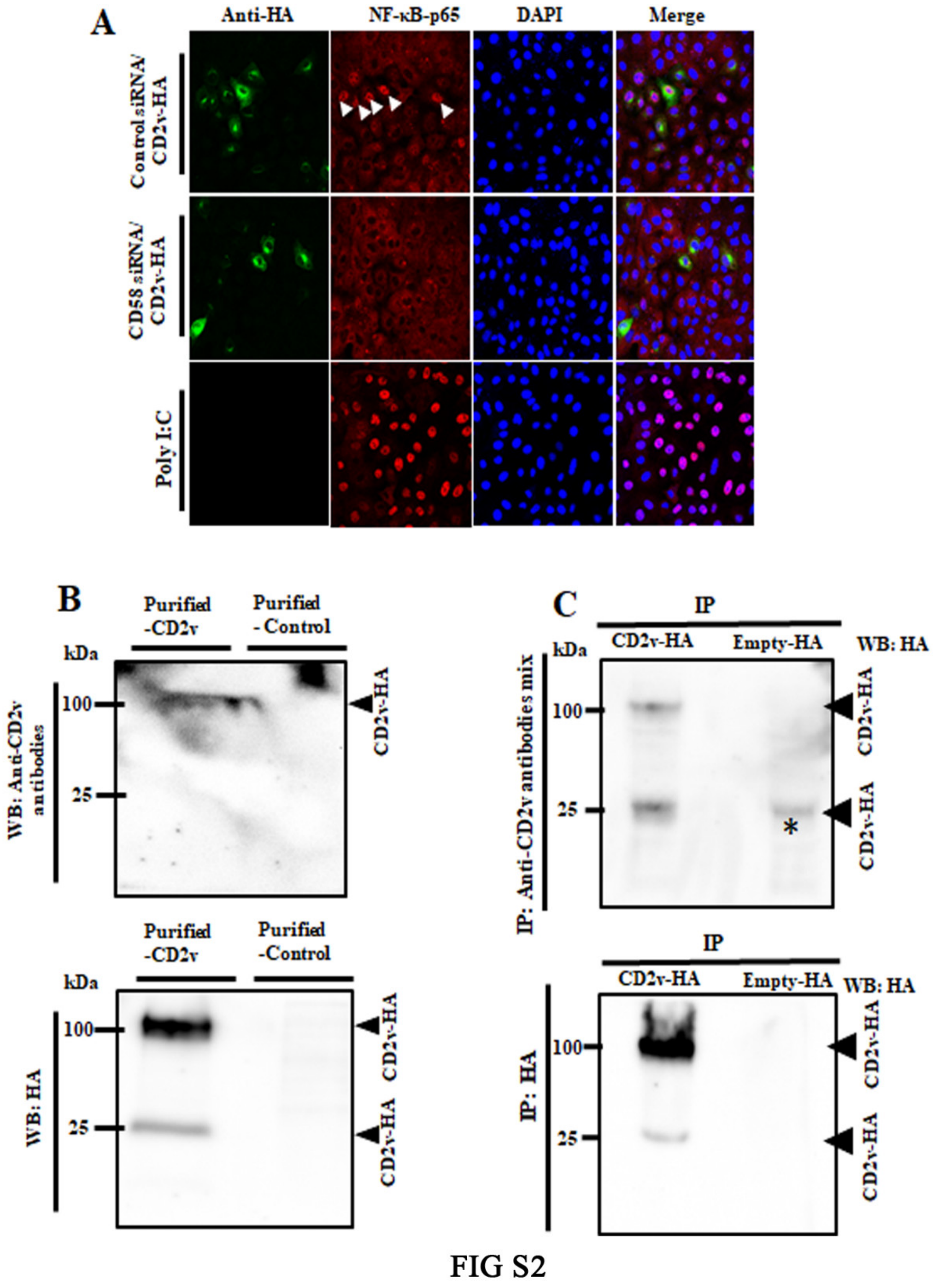

FIG S2

Figure S2. (**A**) Effect of CD58 knock-down on CD2v-induced NF-κB-p65 nuclear translocation. PK15 cells were transfected with CD58 siRNA or siRNA universal negative control for 24 h and then transfected with pCD2v-HA or pEmpty-HA for 3 h. Cells were fixed

and processed for detection of NF-κB-p65 by immunofluorescence. Representative images of NF-κB-p65 nuclear translocation. Green, CD2v; Red, NF-κB-p65; Blue, DAPI. Arrows indicate nuclear NF-κB-p65. (**B**) Monoclonal antibodies generated against ASFV CD2v identifies CD2v in Western blots. Purified CD2v or purified control, were resolved by SDS-PAGE, blotted, and probed with pooled anti-CD2v monoclonal antibodies (A4, C4, C3 and F2; top panel) or probed with anti-HA antibody (control; bottom panel). (**C**) Whole cell extracts were immunoprecipitated with anti-CD2v monoclonal antibodies (top panel) or anti-HA antibody (control; bottom panel) resolved with SDS-PAGE and probed with anti-HA antibody. * denotes light chain band. Results for A-C are representative of three independent experiments.

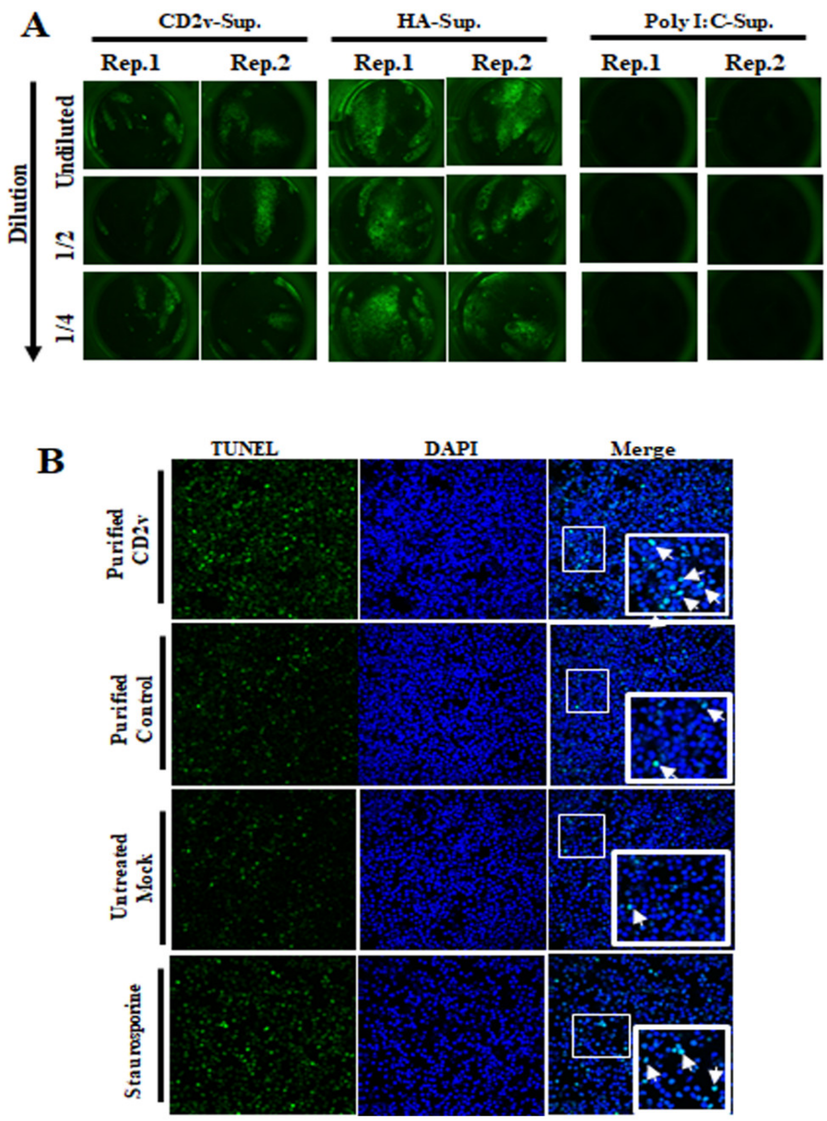

FIG S3

Figure S3. (**A**) Antiviral activity of supernatants from CD2v -treated swine PBMCs. Swine PBMCs were treated with purified CD2v protein or purified control, and supernatants collected 24 h post treatment. Supernatant collected from PK15 transfected with Poly I:C served as a positive control. PK15 grown in 96 well plates were treated with different dilutions of PBMCs supernatants for 24 h and subsequently infected with VSV^{GFP} (50 PFU/well) for 16 h. Shown are representative fluorescence images of PK15 cells fixed at 16 h post VSV^{GFP} infection. Green,

VSV^{GFP}. Rep., denotes technical replicates. (**B**) Representative confocal images of TUNEL assay in swine PBMCs under conditions outlined in Fig. 8D. Green, TUNEL; Blue, DAPI. Insets show magnified areas of the field. Arrows indicate nuclear TUNEL positive cells. Results are representative of three independent experiments.
